# Hereditary deafness carrier screening in 9,993 Chinese individuals

**DOI:** 10.3389/fgene.2023.1327258

**Published:** 2024-01-11

**Authors:** Yanqiu Liu, Lei Wang, Lanlai Yuan, Yaqing Li, Zhengshi Chen, Bicheng Yang, Daqing Wang, Yu Sun

**Affiliations:** ^1^ Jiangxi Maternal and Child Health Hospital, Nanchang, China; ^2^ Dalian Women and Children’s Medical Center (Group), Dalian, China; ^3^ Department of Otorhinolaryngology, Union Hospital, Tongji Medical College, Huazhong University of Science and Technology, Wuhan, China; ^4^ BGI Genomics, BGI-Shenzhen, Shenzhen, China; ^5^ Institute of Otorhinolaryngology, Union Hospital, Tongji Medical College, Huazhong University of Science and Technology, Wuhan, China

**Keywords:** hereditary deafness, carrier screening, reproductive decision making, GJB2, SLC26A4

## Abstract

**Background:** Preconception or prenatal carrier screening plays an important role in reproductive decision-making, but current research on hereditary deafness is limited. This study aimed to investigate the carrier frequencies of common deafness genes in the Chinese population who underwent carrier screening and to follow up on pregnancy outcomes in high-chance couples.

**Methods:** Individual females or couples in preconception or early pregnancy were recruited from two hospitals in China. Carrier screening for common deafness genes in the Chinese population, including the *GJB2* and *SLC26A4* genes, was performed using next-generation sequencing technology. Genetic counseling was provided to subjects before and after testing.

**Results:** Of the 9,993 subjects screened, the carrier rate was 2.86% for the *GJB2* gene and 2.63% for the *SLC26A4* gene. The variant with the highest carrier frequency in *GJB2* was c.235delC (1.89%), and c.919–2A>G (1.08%) in *SLC26A4*. Of the six high-chance couples, four made alternative reproductive decisions (three with prenatal diagnosis and one with preimplantation genetic testing), with consequent termination of the birth of two affected fetuses.

**Conclusion:** These findings confirmed the clinical utility of preconception or prenatal carrier screening for hereditary deafness.

## 1 Introduction

The prevalence of deafness in newborns worldwide is two to three out of every 1,000 births (Alford et al., 2014; Deafness et al., 2023), with more than 50% of the cases attributed to genetic factors (WHO, 2021). In China, the neonatal deafness prevalence rate ranges from 1‰ to 3.47‰ (Yuan et al., 2020). The consequences of untreated deafness, including hereditary deafness, can have a serious impact on a child’s speech, language, education, and social integration (WHO, 2021). Hereditary deafness is a monogenic disorder that follows the Mendelian inheritance pattern and exhibits significant genetic heterogeneity (Yuan et al., 2020). It is estimated that 30% of hereditary deafness is syndromic, and 70% is non-syndromic (WHO, 2021; Li et al., 2022). Of the non-syndromic cases, autosomal dominant and X-linked inheritance account for about 15% and 1%, respectively, while autosomal recessive inheritance is responsible for up to 80% (Li et al., 2022). With the emergence of next-generation sequencing technologies and the decline of sequencing costs, knowledge about the genetic etiology of deafness is rapidly increasing (Li et al., 2022). To date, over 100 related genes are associated with non-syndromic deafness (G and RJH). In the Chinese population, the two primary causative genes for autosomal recessive non-syndromic deafness are the gap junction protein beta 2 gene (*GJB2*; OMIM: 121011) and solute carrier family 26 member 4 (*SLC26A4*; OMIM: 605646) (Ouyang et al., 2009; Wu et al., 2016; Yuan et al., 2020). For the intervention of hereditary deafness, hearing aids can provide good auditory rehabilitation for mild to severe sensorineural hearing loss, but cochlear implantation is preferred for severe to profound hearing loss (Korver et al., 2017). In addition, identification of the genetic etiology can help guide treatment decisions and prevention. For example, in the case of mutations in the OTOF gene, patients with auditory neuropathy spectrum disorders are expected to have preserved auditory nerve function (Korver et al., 2017). If mitochondrial DNA variants are found, an important preventive measure is to avoid using aminoglycosides in relatives (Li et al., 2022). On the other hand, genetic testing can help identify the causative genes, which is essential for accurate genetic counseling, prognosis, and the potential development of possible gene therapy strategies in the future (Kremer, 2019; Li et al., 2022).

It is worth noting that 95% of newborns with deafness identified by newborn hearing screening had hearing parents (Li et al., 2022), suggesting that normal hearing parents who carry the autosomal recessive gene for deafness are at risk of having an affected offspring (Fu et al., 2019). Combined newborn hearing and genetic screening has become widespread and is considered tertiary prevention of deafness (Dai et al., 2019; Deafness et al., 2023), which allows early detection of the molecular etiology and significantly shortens the time to diagnosis and intervention for deafness (Dai et al., 2019). Advancing genetic screening for deafness from the newborn to the prenatal period, namely, carrier screening of pregnant females or couples at risk, together with prenatal diagnosis such as chorionic villus sampling or amniocentesis, can provide secondary prevention of deafness (Gregg et al., 2021; Deafness et al., 2023). Compared to prenatal carrier screening, preconception carrier screening is more recommended (Gregg et al., 2021). Preimplantation genetic testing (PGT) or gamete donation is an option if high-chance couples are detected to be at risk of reproduction (Gregg et al., 2021; Deafness et al., 2023). Thus, preconception carrier screening not only avoids the risk of termination of pregnancy or recurrent abortions, but it can also help couples at risk to have a child with normal hearing, contributing to reducing the incidence of hereditary deafness in newborns (Deafness et al., 2023). Essentially, it is a form of primary prevention for deafness (Deafness et al., 2023). A couple who are both confirmed carriers of the same autosomal recessive disorder has a 25% probability of having an affected child with each pregnancy. If the mother carries an X-linked recessive disease, the male offspring of the couple has a 50% probability of being affected (Van Steijvoort et al., 2020). In 2013, the first study of carrier screening for the deafness gene in women of childbearing age showed that eight out of nine couples carrying variants in the same deafness gene received the prenatal diagnosis and subsequently prevented the birth of one affected fetus (Yin et al., 2013). Accordingly, preconception or prenatal carrier screening for hereditary deafness enables those screened to consider their reproductive risk and promotes additional reproductive options (Henneman et al., 2016; Gregg et al., 2021; Best et al., 2023). However, studies of preconception or prenatal carrier screening for hereditary deafness are limited, and more investigations are needed.

There is no consensus on which genes for hereditary deafness should be tested for preconception or prenatal carrier screening. Given the high carrier rates of the *GJB2* and *SLC26A4* genes in the Chinese population (Zhang et al., 2023) and following the latest recommendations of the American College of Medical Genetics and Genomics in 2021 (Gregg et al., 2021), *GJB2*-associated autosomal recessive deafness-1A (DFNB1A; OMIM: 220290) and *SLC26A4*-associated autosomal recessive deafness-4 (DFNB4) with enlarged vestibular aqueduct (OMIM: 600791) or Pendred syndrome (PDS; OMIM: 274600) were included in this study for preconception or prenatal carrier screening. This study aimed to perform carrier screening in individual females or couples before or early in pregnancy to determine carrier rates of the *GJB2* and *SLC26A4* genes and their variants. In addition, we provided genetic counseling to all subjects and followed up on reproductive decision-making, pregnancy outcomes and neonatal health status in high-chance couples.

## 2 Materials and methods

### 2.1 Subjects and study design

A total of 9,993 subjects, including 1,783 couples, were enrolled in this study from Jiangxi Maternal and Child Health Hospital (from March 2020 to July 2023) and Dalian Woman and Children’s Medical Center (from August 2020 to July 2023) in China. These subjects attended an expanded carrier screening program (including preconception or prenatal) for 155 monogenic disorders (Supplementary Table S1). The study focused on preconception or prenatal carrier screening for hereditary deafness of our interest, consisting of DFNB1A (*GJB2* gene) and DFNB4 or PDS (*SLC26A4* gene). The criteria for inclusion were: no family history of hereditary deafness without the phenotype of deafness; couples of childbearing age preparing for pregnancy or with gestational weeks of less than sixteen; couples seeking a healthy child through assisted reproductive technology; and couples of close consanguinity. Those with a family history of hereditary deafness or gestational age of 16 weeks or greater were excluded. Genetic counseling prior to testing was provided to all subjects, including the purpose, procedure, benefits, risks, limitations, and possible results of carrier screening, to enable patients to make an informed and autonomous decision about whether or not to undergo screening (Gregg et al., 2021; Li et al., 2022). All subjects signed an informed consent form. Approval for this study was granted by the Institutional Review Board of BGI.

Two strategies were used for carrier screening: sequential and concurrent screening. In sequential screening, females were tested first, and their partners were recalled if they carried at least one variant in the autosomal recessive deafness gene. In concurrent screening, the couples underwent genetic testing at the same time. After testing, subjects were provided genetic counseling, including interpretation of results (even uncertain and negative results), information on prognosis, chance of having affected children in the future, accessible reproductive options, and remaining risk (Gregg et al., 2021; Li et al., 2022). Additionally, high-chance couples were given recommendations such as prenatal diagnosis, PGT or gamete donation.

### 2.2 Selection of hereditary deafness diseases and sequencing

DFNB1A (OMIM: 220290) and DFNB4 with enlarged vestibular aqueduct (OMIM: 600791) or PDS (OMIM: 274600) were selected for preconception or prenatal carrier screening in this study.

The peripheral blood samples of the subjects were collected in 2–5 mL, and genomic DNA was extracted. The target regions (exons and splice site sequences of genes) in genomic DNA were captured using a set of oligonucleotide probes, followed by detection using high-throughput sequencing technology. Bioinformatic analysis of the assay results was used to identify the likely pathogenic or pathogenic variants of the target genes. According to the guidelines of the American College of Medical Genetics and Genomics (Richards et al., 2015), the screened loci were interpreted and reports were generated.

### 2.3 Follow up

High-chance couples were followed up on their reproductive decision-making (e.g., prenatal diagnosis and PGT), pregnancy outcomes, and neonatal health conditions.

### 2.4 Statistical analyses

Numbers (percentage) were reported for categorical variables, and means (standard deviation) for continuous variables. All statistical analyses were performed with R version 4.2.3.

## 3 Results

### 3.1 Baseline characteristics of enrolled subjects

**FIGURE 1:**
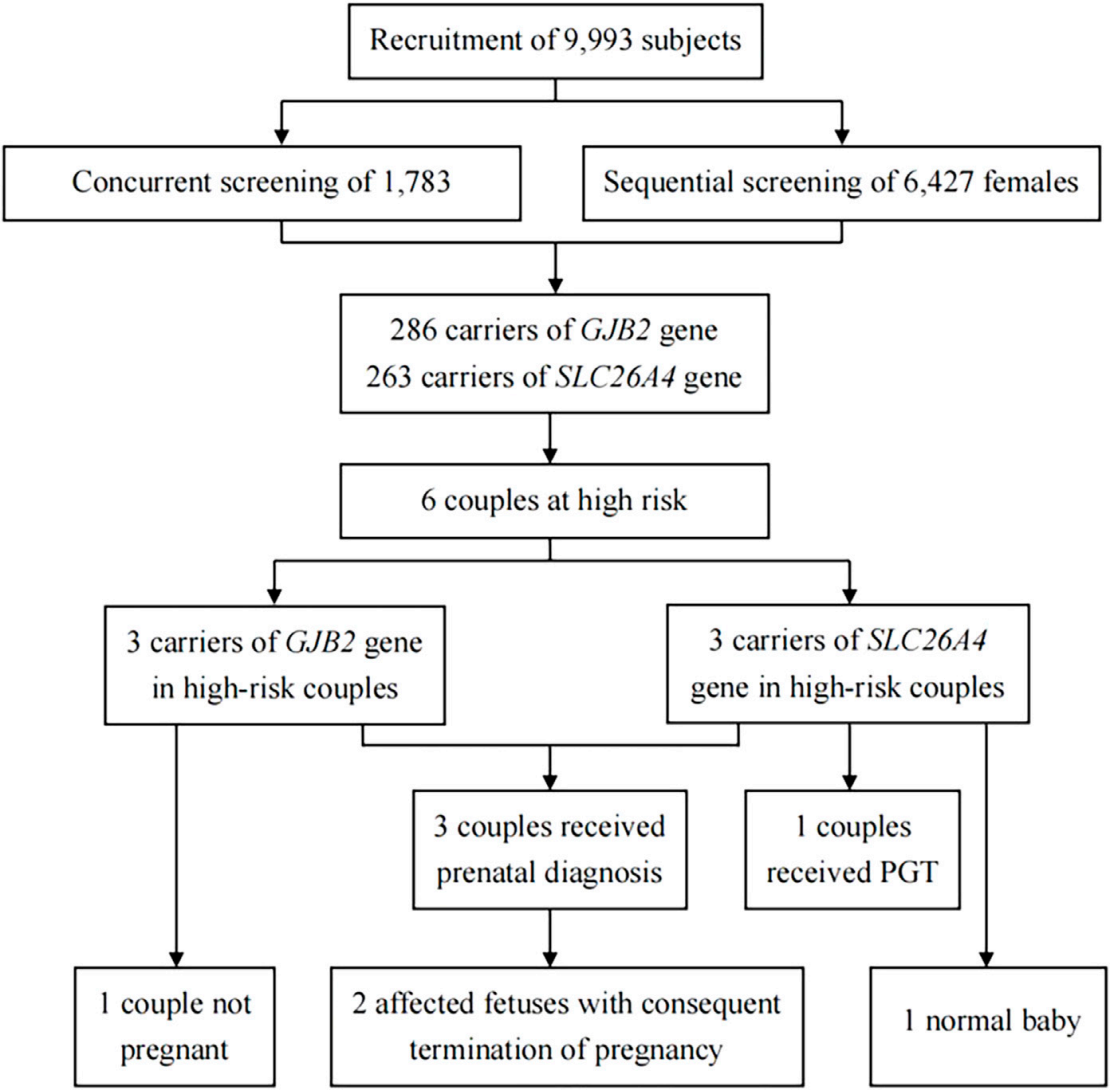
Strategy and flowchart for carrier screening. PGT, preimplantation genetic testing.

Of the 9,993 subjects recruited for this study, 82.2% (n = 8,210) were females, and 17.8% (n = 1,783) were males (i.e., 17,83 couples and 6,427 females). A greater number of 69.1% (n = 6,903) were from Jiangxi Province, and the remaining 30.9% (n = 3,090) were from Liaoning Province. Figure 1 shows the strategy and flowchart for carrier screening in this study.

### 3.2 Carrier frequencies of genes and variants associated with deafness

In total, 549 subjects were identified as carriers of deafness-related genes, yielding a carrier frequency of 5.49%. As shown in Table 1, there were 286 carriers of the *GJB2* gene with a frequency of 2.86%, and 263 carriers of the *SLC26A4* gene with a frequency of 2.63%. Table 2 shows the top three carrier frequencies of variants in the *GJB2* and *SLC26A4* genes. The top three variants in the *GJB2* gene were c.235delC with a frequency of 1.89%, c.299_300del with a frequency of 0.58%, and c.176_191del with a frequency of 0.11%. For the *SLC26A4* gene, they were c.919–2A>G with a frequency of 1.08%, c.2168A>G with a frequency of 0.21%, and c.1229C>T with a frequency of 0.20%. In addition, six subjects carried compound heterozygotes with a combination of variants in the *GJB2* and *SLC26A4* genes, and three subjects carried compound heterozygous variants in the *SLC26A4* gene (Supplementary Tables S2 and S3). The detailed variant spectra of the *GJB2* and *SLC26A4* genes were shown in Supplementary Table S2 and Supplementary Table S3.

**TABLE 1:** Carrier frequencies of deafness genes among 9,993 subjects.

OMIM gene	Gene	RefSeq number	OMIM phenotype	Type of hereditary deafness	Count	Carrier frequency (%)
121,011	*GJB2*	NM_004004.6	220,290	DFNB1A	286	2.86
605,646	*SLC26A4*	NM_000441.2	600,791/274,600	DFNB4/PDS	263	2.63
-	-		-	-	549	5.49

GJB2, gap junction protein beta 2; SLC26A4, solute carrier family 26 member 4; DFNB1A, nonsyndromic deafness, autosomal recessive 1A; DFNB4, deafness autosomal recessive 4; PDS, pendred syndrome.

**TABLE 2:** The top three carrier frequencies of variants in the GJB2 and SLC26A4 genes among 9,993 subjects.

Gene	Variant	SNP ID	Count	Carrier frequency (%)
*GJB2*				
	c.235delC (p.Leu79fs)	rs80338943	189	1.89
	c.299_300del (p.His100fs)	rs111033204	58	0.58
	c.176_191del (p.Gly59fs)	rs750188782	11	0.11
*SLC26A4*				
	c.919–2A>G	rs111033313	108	1.08
	c.2168A>G (p.His723Arg)	rs121908362	21	0.21
	c.1229C>T (p.Thr410Met)	rs111033220	20	0.20

SNP, single nucleotide polymorphism.

### 3.3 Couples at high-chance and status of follow-up

As shown in Table 3, there were six couples (two pregnant and four not pregnant when recruited) carrying variants in the same *GJB2* or *SLC26A4* gene. Among the high-chance couples carrying the *GJB2* gene, two couples underwent prenatal diagnosis, resulting in the termination of their pregnancy due to the fetus being affected; and one was not pregnant at the time of follow-up. Of the high-chance couples carriers of the *SLC26A4* gene, one was pregnant during the follow-up and underwent prenatal diagnosis with the fetus detected as heterozygous for the c.919–2A>G variant; one was performing preimplantation genetic testing to reduce the risk of affected offspring; and the remaining one did not receive a prenatal diagnosis but gave birth to a baby with normal hearing.

**TABLE 3:** High-chance couples and follow-up status.

Gene	Gender	Variant	Prenatal diagnosis/outcomes of fetus	Affected fetus	Pregnancy outcome
*GJB2*					
	Female	c.299_300del (p.His100fs)	Yes/-	Yes	Termination
	Male	c.235delC (p.Leu79fs)			
	Female	c.235delC (p.Leu79fs)	Yes/-	Yes	Termination
	Male	c.235delC (p.Leu79fs)			
	Female	c.176_191del (p.Gly59fs)	No/-	-	Not pregnant
	Male	c.235delC (p.Leu79fs)			
*SLC26A4*					
	Female	c.2168A>G (p.His723Arg)	Yes/heterozygous variant of c.919-2A>G	No	-
	Male	c.919-2A>G			
	Female	c.919-2A>G	No/-	Three blastocysts obtained by PGT await for testing	-
	Male	c.1595G>T (p.Ser532Ile)			
	Female	c.1692dup (p.Cys565fs)	No/-	No	A normal baby
	Male	c.1226G>A (p.Arg409His)			

PGT, preimplantation genetic testing.

## 4 Discussion

In this study, preconception or prenatal carrier screening for the deafness genes *GJB2* and *SLC26A4* was performed in 9,993 individuals from China, with frequencies of 2.86% and 2.63%, respectively. The most common variant in *GJB2* was c.235delC, while in *SLC26A4*, it was c.919–2A>G. In addition, three out of six high-chance couples underwent prenatal diagnosis with the avoidance of the birth of two fetuses with hereditary deafness, and one out of six chose PGT to reduce the risk of offspring being affected. The carrier frequencies of common deafness genes and their variants in the Chinese population identified in this study might provide data support for future research and clinical genetic counseling. Moreover, this study suggested the importance of preconception or prenatal carrier screening for hereditary deafness to assess reproductive risk and guide reproductive decision-making.

The carrier frequency of the *GJB2* gene in this study was 2.86%, which was slightly higher than the frequency of 1.66% found in a preconception or prenatal expanded carrier screening study of 10,476 couples (i.e., 20,952 individuals) from southern China (Zhao et al., 2019). ​However, a recent study of 3,555,336 neonates from the Chinese Newborn Concurrent Hearing and Genetic Screening cohort reported a similar carrier rate, with an overall *GJB2* carrier rate of 2.53% (Zhang et al., 2023). Consistent with previous studies in the Chinese population (Dai et al., 2019; Fu et al., 2019; Zhang et al., 2023), our study found the top three carrier frequency variants of the *GJB2* gene to be c.235delC (1.89%), c.299_300del (0.58%) and c.176_191del (0.11%). A recent study conducted concurrent hearing and genetic screening for 180,469 newborns in Beijing showed that the frequencies of c.235delC, c.299_300del, and c.176_191del were 1.80%, 0.50%, and 0.12%, respectively (Dai et al., 2019). In another study of newborns, the carrier frequencies were 1.95%, 0.48%, and 0.11%, respectively (Zhang et al., 2023). Variants in the *GJB2* gene are the leading cause of autosomal recessive non-syndromic deafness in the Chinese population (Ouyang et al., 2009; Wu et al., 2016). *GJB2* encodes the gap junction protein, which is essential for the maintenance of cochlear homeostasis through the recycling of potassium in the inner ear (Mammano, 2019; Sheffield and Smith, 2019). The deafness associated with *GJB2* is sensorineural and varies in severity from mild to profound (Li et al., 2022). Generally, it is present at birth (Li et al., 2022).

The second most common cause of autosomal recessive non-syndromic deafness in the Chinese population is the *SLC26A4* gene (Ouyang et al., 2009; Wu et al., 2016). ​​We found that the frequency of the *SLC26A4* gene in preconception or prenatal carrier screening was 2.63%, which was comparable to the previously reported carrier rate of 2.05% in 3,555,336 newborns in China (Zhang et al., 2023), but higher than the 1.59% in 10,476 couples (Zhao et al., 2019). Furthermore, the three most common variants of the *SLC26A4* gene were c.919–2A>G with a frequency of 1.08%, c.2168A>G with a frequency of 0.21% and c.1229C>T with a frequency of 0.20%. Similarly, these variants were the top three in frequency among 3,555,336 newborns in China, with frequencies of 1.32%, 0.24%, and 0.12%, respectively (Zhang et al., 2023). The *SLC26A4* gene encodes the pendrin protein, which is expressed in the inner ear, thyroid and kidney (Sheffield and Smith, 2019; Honda and Griffith, 2022). *SLC26A4* is the gene causing DFNB4 and PDS, whose related phenotypes are described as inner ear malformations, hearing impairment, vestibular dysfunction, and thyroid abnormalities (Honda and Griffith, 2022). The hearing impairment associated with *SLC26A4* is typically fluctuant or progressive sensorineural (Honda and Griffith, 2022).

Carrier screening can provide an opportunity for individuals or couples to know their risk and consider available reproductive options at a preconception or prenatal stage, which demonstrates its clinical utility (Gregg et al., 2021). If identified before pregnancy, high-chance couples have the options of preimplantation genetic testing, gamete or embryo donation, prenatal diagnosis (chorionic villus sampling or amniocentesis), and adoption, followed by decision-making on whether to continue the pregnancy (Gregg et al., 2021). If identified during pregnancy, however, the option is a prenatal diagnosis followed by decisions on whether to prepare for medical care after an affected child is born or to terminate the pregnancy (Gregg et al., 2021). Of the six high-chance couples, except for one who did not become pregnant, four made an alternative reproductive choice (three underwent prenatal diagnosis and one chose PGT), resulting in the termination of the birth of two affected fetuses. Therefore, our results supported the clinical utility of preconception or prenatal carrier screening for hereditary deafness.

There were several limitations to the present study. Firstly, the sample was underrepresented because of the inclusion of only subjects from Jiangxi and Liaoning provinces in China. In addition, detailed information on ethnicity was not available for this study. Secondly, although the *GJB2* and *SLC26A4* genes are the major genetic causes of autosomal recessive non-syndromic deafness in the Chinese population, other related deafness genes that were not screened might have been missed. The discrepancy between the carrier frequencies obtained in this study and those in other studies may be due to variations in the representativeness of the samples (e.g., ethnicity and geographical location) and the coverage of the genes and their variants screened. Therefore, future studies should optimize carrier screening for deafness genes and their variants suitable for different ethnic backgrounds and geographical locations. Finally, complete information on pregnancy outcomes in high-chance couples could not be tracked.

## 5 Conclusion

In conclusion, this study performed preconception or prenatal carrier screening for the common deafness genes *GJB2* and *SLC26A4* in 9,993 individuals from China, showing carrier frequencies of 2.86% and 2.63%, respectively. In addition, four out of six high-chance couples made alternative reproductive decisions, followed by the prevention of the birth of two affected fetuses. These findings confirmed the clinical utility of preconception or prenatal carrier screening for hereditary deafness.

## Data Availability

The raw data supporting the conclusions of this article will be made available by the authors, without undue reservation.
